# Vergence Fusion Sustaining Oscillations

**DOI:** 10.16910/jemr.14.1.4

**Published:** 2021-06-28

**Authors:** John Semmlow, Chang Yaramothu, Mitchell Scheiman, Tara L. Alvarez

**Affiliations:** Rutgers University; New Jersey Institute of Technology; Salus University

**Keywords:** vergence, slow component, fusion sustaining component, feedback control, oscillations

## Abstract

Introduction: Previous studies have shown that the slow, or fusion sustaining, component of disparity vergence contains oscillatory behavior as would be expected if fusion is sustained by visual feedback. This study extends the examination of this behavior to a wider range of frequencies and a larger number of subjects.

Methods: Disparity vergence responses to symmetrical 4.0 deg step changes in target position were recorded in 20 subjects. Approximately three seconds of the late component of each response were isolated using interactive graphics and the frequency spectrum calculated. Peaks in these spectra associated with oscillatory behavior were identified and examined.

Results: All subjects exhibited oscillatory behavior with fundamental frequencies ranging between 0.37 and 0.55 Hz; much lower than those identified in the earlier study. All responses showed significant higher frequency components. The relationship between higher frequency components and the fundamental frequency suggest may be harmonics. A correlation was found across subjects between the amplitude of the fundamental frequency and the maximum velocity of the fusion initiating component probably due to the gain of shared neural pathways.

Conclusion: Low frequency oscillatory behavior was found in all subjects adding support that the slow, or fusion sustaining, component is mediated by a feedback control.

## Introduction

**Figure 1:**
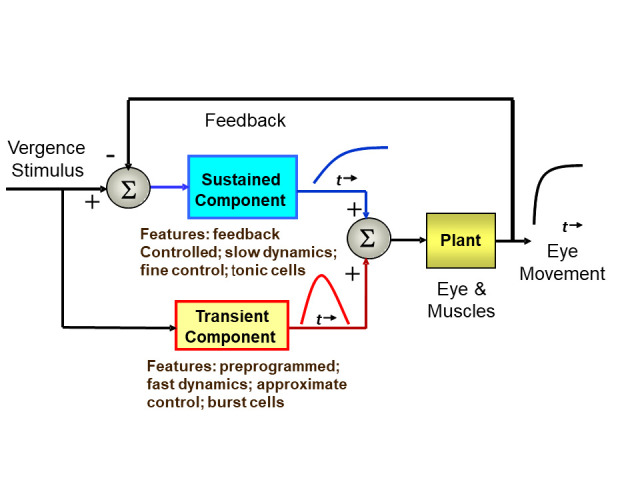
A schematic model of the dual-mode control strategy showing pathways for fusion initiating and fusion sustaining components (from Semmlow et al., 21).

To attain high velocity responses, motor movements often rely on open-loop, or preprogrammed control strategies. However, such movements have limited accuracy. Feedback control can produce extremely accurate responses, but if delays are present within the feedback loop, then response velocity must be reduced to keep the system stable. Both version and vergence control systems achieve speed and accuracy by combining the two strategies. In version, the two control strategies manifest as separate movements: preprogrammed saccades and feedback-controlled pursuit movements. In vergence, the two control components are less obvious as they merge into a single coordinated response. Nonetheless, considerable evidence supports a “dual mode” control strategy ( 25; 26; 27; 23; 24; 22; 13; 11; 2; 4; ). This approach consists of an open-loop, fusion initiating component that enhances initial dynamics followed by a fusion sustaining component driven by visual and internal feedback to slowly bring the response to the final position (Alvarez et al. 2000). We favor the term “dual-mode” to describe this strategy as opposed to “pulse-step” as it emphasizes the difference in control strategies: open-loop versus feedback control. Evidence of a dual-mode strategy for the control of disparity eye movement has been provided from both behavioral studies ( 15; 25; 26; 27; 11; 2; 16 ) and neurophysiological studies (17; 18). A schematic representation of vergence control is summarized in Figure 1.

The fusion sustaining component has not been as well-studied as the fusion initiating component. The assumption that it is feedback controlled is based on the fact that sustained vergence has high positional accuracy (a few minutes of arc Ogle, 19) which would require feedback. It would be impossible to achieve such accuracy from a noisy and variable neurological control system without the use of visual feedback. There may also be an internal feedback pathway(s) that bypasses some of the visual delays to improve stability. In the fusion initiating component of vergence, efference copy signals have been reported to improve stability (Alvarez et al. 2000).

We know that feedback control systems will exhibit instabilities in the form of oscillatory behavior if the loop gain or loop delay exceed certain limits. Given that the time delay of a typical vergence response ranges between approximately 0.18 to 0.24 sec. (6) and that the loop gain must be high to achieve small fixation errors, feedback system analysis predicts oscillations will occur during the fusion sustaining response. The nature of these oscillations, particularly their frequencies and amplitudes have direct implications on the neural control components that sustain vergence fixation. Oscillatory behavior is best identified by determining the frequency spectrum of the data usually implemented by applying the Fourier Transform to a data segment as previously done by our group (21). In a study involving 8 subjects, they found oscillatory behavior in all subjects with a fundamental frequency (i.e., lowest frequency) ranging between 1.2 and 1.9 Hz and a number of higher frequency peaks. Spectra from different subjects varied somewhat in fundamental frequencies, component magnitude and the number of spectral peaks. Higher frequency components (those above the fundamental frequency) had frequencies that were related to the fundamental frequency. This indicates that the higher frequency components were harmonics of the fundamental oscillation.

In their original study of fusion sustaining oscillations, Semmlow and colleagues (21) were limited to vergence step responses recorded over a 2 sec time frame. After eliminating the fusion initiating response, defined as the transient portion of the response, the length of fusion sustaining late component available for analysis was only 1.2 sec, at most. This limits the lowest frequency component that can be identified thought spectral analysis to approximately 0.8 Hz. In this study, we extend the data collection period to 4 sec providing approximately 3.2 sec of fusion sustaining component and allowing us to search for lower frequency components. A data acquisition time of 3 or more seconds can identify frequencies as low as 0.33 Hz.

## Methods

### Subjects

The inclusion criteria for this study were young adults between the ages of 18 to 35 with normal binocular vision and accommodation. Twenty binocularly normal young adult subjects (3 females) between 18 and 26 years of age (21.1 ±2.5 years) participated in this study. All subjects signed written informed consent approved by the New Jersey Institute of Technology Institution Review Board. An oculomotor examination was conducted by an optometrist who is one of the authors (MS). Exclusion criteria included those with traumatic brain injury, any disease known to impact the accommodative or vergence systems, anyone with presbyopia or subjects who had myopia of more than –5D or those with hyperopia more than +2D.

### Clinical Examination

The oculomotor assessment included the following measurements: best-corrected visual acuity at distance, near point of convergence (NPC), positive and negative fusional vergence, eye alignment at 4m and 40 cm using the unilateral cover test and prism and alternate cover test, stereopsis, vergence facility, and amplitude of accommodation. These measures were performed using previously published protocols and can be assessed at http://optometry.osu.edu/research/CITT /4363.cfm.

Briefly, NPC was measured from the bridge of the nose using the near point rule where a high acuity target 20/30 size column letters (Gulden Fixation Stick no. 15302) was moved towards the person. The distance from the bridge of the nose to the visual target position when the subject reported diplopia was recorded as NPC. Positive and negative fusional vergence was measured with a horizontal prism bar (Gulden B-15 horizontal prism bar—1∆ to 45∆). The subject viewed a 20/30 size column of letters held stationary at 40 cm along the subject’s midsagittal plane. The base-out prism at which the person saw blur or diplopia which ever occurred first was the positive fusional vergence (PFV). Negative fusional vergence (NFV) was similar measured but using base-in prism. Vergence facility was recorded using a 12∆ base-out with 3∆ base-in prism set on a rod. The subject would view the 20/30 column of letters also held stationary at 40 cm away along the subject’s midline. The participant would report when they saw the visual target single and clear and then the examiner would change the other prism. The number of cycles (viewing the base-out and then the base-in) of the prism were recorded within 1 minute.

Amplitude of accommodation was measured monocularly for the right eye where the left eye was occluded, and the subjects viewed the 20/30 single column of letters. Stereopsis was assessed using the Randot Stereo Test. All subjects had stereopsis of better than 70 arcseconds. Acuity was assessed and all subjects were properly refracted to 20/20 vision for all eye movement experiments. Near (measured at 40cm) and far (measured at 6m) heterophoria was assessed using the alternate cover test.

The full optometric exam parameters for all subjects are found in Table 1 below indicating that all participants had normal binocular vision assessed by an Optometrist.

**Table 1:** Optometric Exam Parameters

Parameter	Average ± SD	Range
NPC (cm)	3.9 ± 1.1	2 to 5.5
NFV (∆)	14.5 ± 2.5	10 to 20
PFV (∆)	28 ± 6.6	16 to 40
Vergence facility (cycles/min)	16 ± 5.3	10 to 29
Amplitude of Accommodation (D)	9.3 ± 1.8	8 to 15
Stereopsis (sec of arc)	33 ± 14.5	20 to 70
Near Heterophoria (∆)	2 exo±2.3	-7.5 exo to 2 eso
Far Heterophoria (∆)	0 ± 0.5	-0.5 exo to 1 eso

### Eye Movement Instrumentation/ Calibration

Left and right-eye movements were recorded using an infrared video-based ISCAN RK-826PCI (Burlington, MA) binocular eye tracker with a reported accuracy of 0.3^º^ over a ±20^º^ horizontal and vertical range. Subjects were centered in front of two partially reflective mirrors and viewed visual stimuli on two computer screens. These computer screens were placed 40 cm from the subject and arranged as a haploscope for a constant accommodative demand of 2.5D. Visual stimuli were controlled using a custom software package (9). A strength of our proprietary system is that the raw eye movements were digitized using a 12-bit digital acquisition (DAQ) hardware card (National Instruments 6024 E series, Austin, TX, USA) at 500 Hz. This is important because our visual stimuli presentation and eye movement recording were controlled by our custom LabVIEW program that would allow temporal analyses.

Eye movement responses were calibrated using 6 monocular targets at 1, 3, and 5 deg. in each eye separately. Monocular calibration reduces the influence of fixation disparity (14) which is important for the accuracy of the amplitude of the vergence movement but has minimal impact the frequency analysis. Using the calibration data, the eye position traces were converted to degree of rotation for each eye trace. Vergence responses were computed as the difference between left and right eye movement responses. Convergence was plotted as positive.

### Visual Stimuli

A range of both symmetrical convergence and divergence stimuli were presented randomly to reduce anticipatory cues (1), but only responses to 4.0 deg. step stimuli (from 4.0 deg to 8.0 deg binocular vergence angular demand) were used in this analysis. This stimulus range was selected because it was comfortable for most subjects and produces more responses free of saccades or other artifacts. From 4 to 15 artifact-free recordings were obtained from each of the 20 subjects.

### Analysis

**Figure 2:**
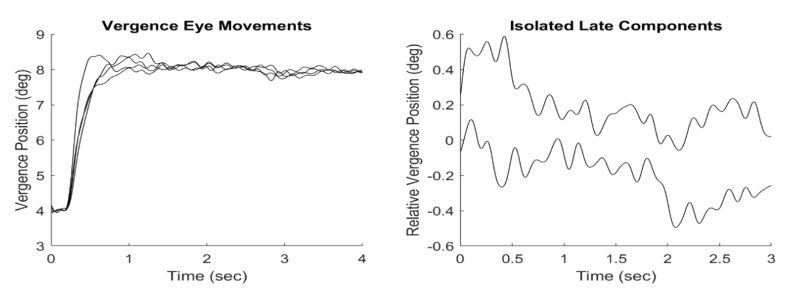
Left graph: An ensemble of 4 vergence eye movements in response to a step stimulus from 4.0 to 8.0 deg convergent. Right graph: Fusion sustaining components isolated from 2 of the responses on the left showing the last 3 seconds of the recorded responses.

A typical ensemble of vergence eye movements is shown in Figure 2, left graph. The fusion sustaining components of these responses were isolated from the late segment of each response using a velocity-based criterion as described in Semmlow et al. (21). The frequency spectra obtained from these isolated spectra using the Fourier Transform as also detailed in Semmlow et al. (21). Peak frequencies and amplitudes were obtained by identifying peaks in the spectra curves. The identification of the fusion sustaining component need only be approximate as slight variations in timing will not affect the results (magnitude spectra are insensitive to time-shifts). While the time responses shown in Figure 2 were lowpass filtered to improve clarity, the spectra were obtained from unfiltered data as any high frequency noise would not affect the lower frequencies of interest in this study. A Pearson’s correlation coefficient (*r-*value) was used to assess correlations.

## Results

**Figure 3:**
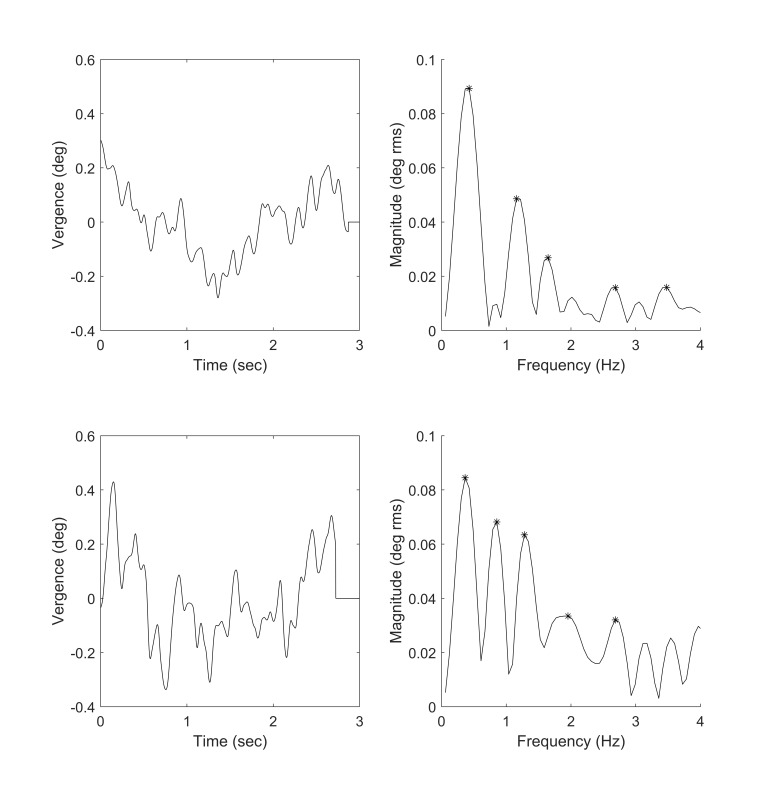
The plots on the left show two examples of fusion sustaining components isolated from vergence responses. The plots on the right show the magnitude frequency spectra associated with these components. The ‘*’ marks indicate frequency peaks.

**Figure 4:**
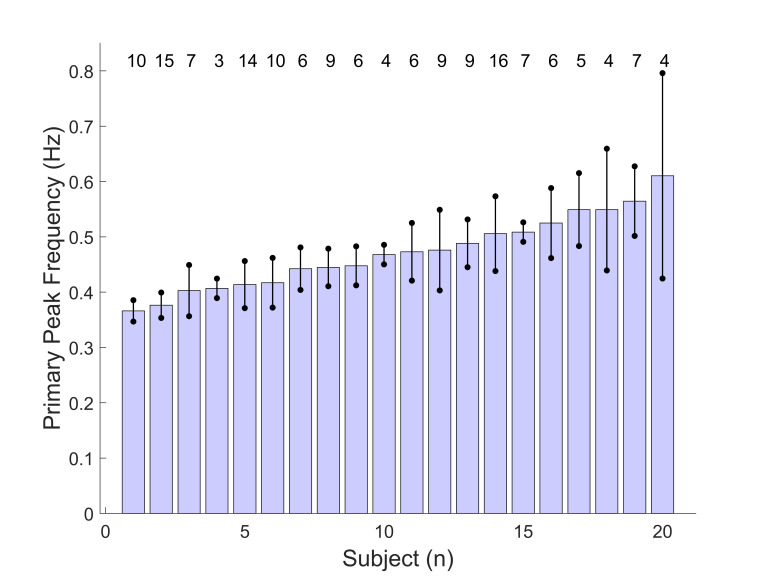
Bar graph showing the fundamental frequencies, in Hz, found for the 20 subjects. The filled section of each bar indicates the frequency (units of Hz) averaged over all responses with error bars indicating one standard deviation Top numbers indicate number of responses.

The plots in Figure 3 show two isolated fusion sustaining components and their associated magnitude frequency spectra. While individual spectra varied considerably, they have some common features including a large fundamental frequency below 0.5 Hz and a number of higher frequency peaks. The fundamental frequencies for the 20 subjects are summarized in the bar graph of Figure 4 (next page) and subjects have been arranged in order of increasing frequency.

As shown in Figure 4 (below), the fundamental frequencies found in all responses averaged between 0.37 and 0.55 Hz and indicate the presence of a low frequency oscillatory process. These oscillations are likely due to the slow component feedback system.

**Figure 5:**
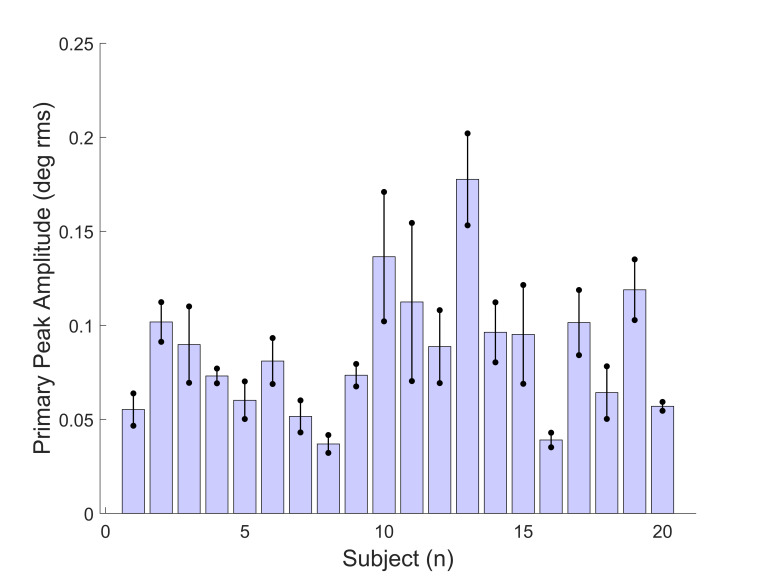
The amplitude of the fundamental frequency spectral peaks presented in the same order and format as in Figure 4. Averaged peak amplitudes varied widely between 0.04 and 0.18 deg rms.

The amplitudes of the fundamental frequency peaks varied widely across subjects as shown in Figure 5. The average spectral peaks amplitude ranged from 0.05 to 0.16 deg rms. The variation in amplitude is much larger than that of frequency.

**Figure 6:**
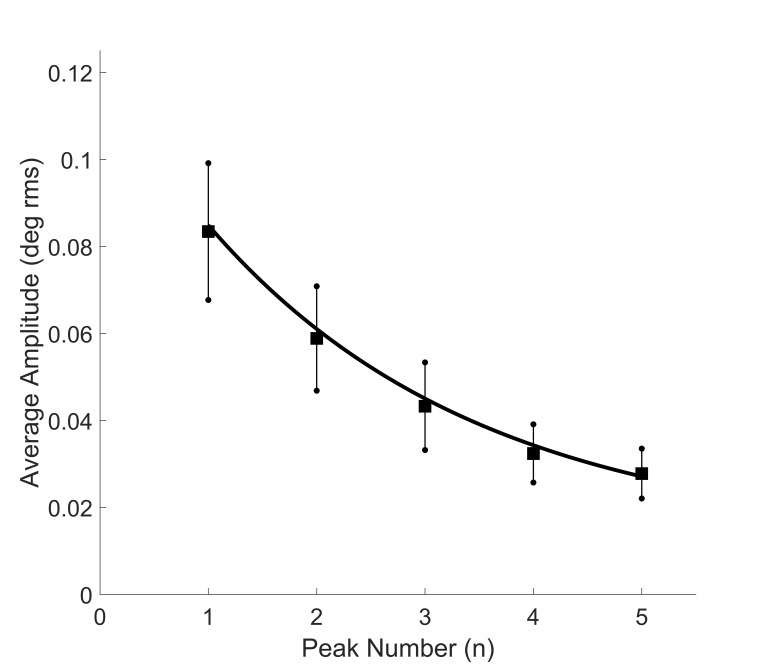
Mean amplitudes (squares) of the first 5 spectral peaks averaged across all subjects. The large standard deviations reflect the large variability in the amplitude of the oscillation. A least squares algorithm was used to fit an exponential to the data.

Figure 6 shows the amplitudes of the first 5 spectral peaks averaged across all responses and all subjects. The large standard deviations reflect the large variability of oscillatory amplitude, both between and within subjects. Note that the average amplitudes decrease exponentially as oscillatory frequency increases.

**Figure 7:**
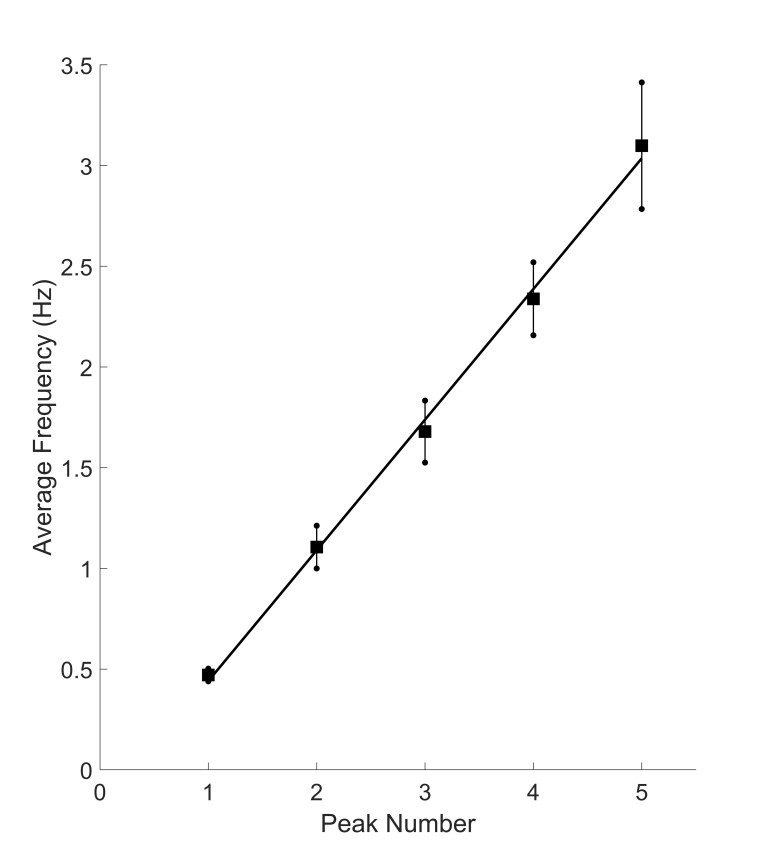
Average frequency in Hz of the fundamental frequency (n = 1) and the first 4 harmonics averaged across all subjects. The small standard deviations reflect the relative constancy of the fundamental (i.e., first) frequency across the subjects. Note that the middle three peaks fall very close to a straight line (dotted blue line).

The frequencies of the additional peaks are also related to the fundamental frequency. Figure 7 shows peak frequencies averaged across all responses and all subjects for the first five peaks as was done for peak amplitude in Figure 6. These additional peaks line along a straight line. The standard deviations for frequency are much less that those for amplitude (Figure 6) reflecting the reduced variability for this spectral feature.

Figure 7 shows a strong relationship between peak frequencies suggesting the various spectral peaks are harmonics of the fundamental frequencies. Such harmonics would be generated by nonlinearities in the motor pathway as indicted by the model in Semmlow and colleagues (21). Harmonic frequencies are multiples of the fundamental frequency:

**(Eq. 1):**
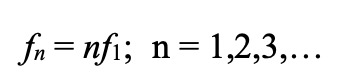


where *f*_1_ is the fundamental frequency, *f_n_* are the harmonic frequencies, and *n* is the harmonic number (the order in which the peak occurs in the spectrum).

**Figure 8:**
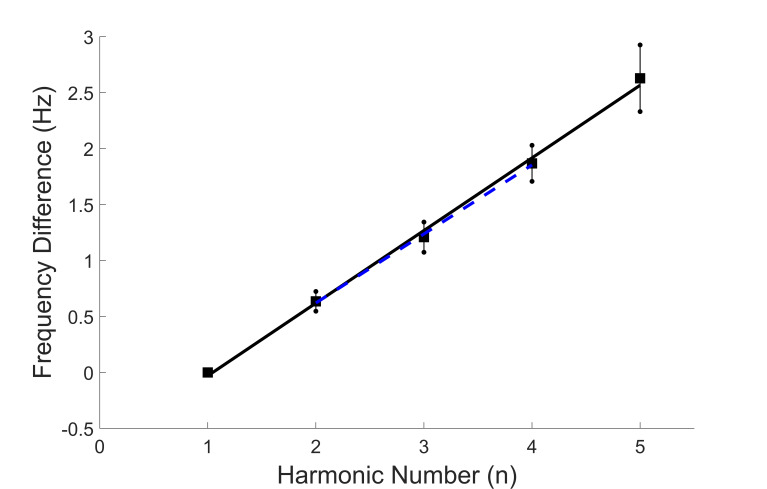
Average frequency in Hz of the fundamental frequency (n = 1) and the first 4 harmonics plotted as in Figure 7 except the frequencies are normalized to f_1_, the fundamental frequency. The slope of the first 3 harmonics is 0.62 Hz averaged across all subjects.

Eq. 1 indicates that if the higher frequency peaks are harmonics of the fundamental frequency, f_1_, then f_n_ should be proportional to n with a constant of proportionality of f_1_. That is, a plot of f_n_ as a function against n, should result in a straight line with a slope of f_1_ and intercept of 0.0 (f_n_ = 0 when n = 0). In Figure 8 we show a plot similar to that in Figure 7, but the frequencies are normalized to the value of f_1_; i.e., f_1_ is subtracted from the frequency values. This is to account for the inter-subject differences in f_1_. The slope of the curve in Figure 8 is 0.65, higher than the average value of f_1_ which is 0.47. Note that the first higher frequency peaks (n = 2 to 4) fall very close to a straight line. The slope of this line somewhat closer to f_1_ at 0.62. Possible causes of the discrepancy in fundamental frequency are described in the Discussion.

**Figure 9:**
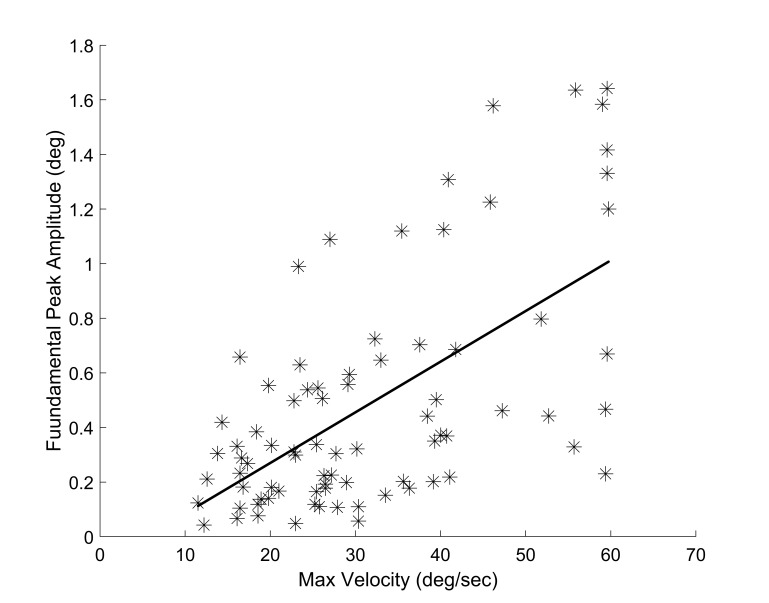
Relationship between the amplitude of the fundamental frequency peak in the fusion sustaining component and the maximum velocity of the fusion initiating component for all responses from all subjects.

A correlation analysis was conducted between peak amplitudes and frequencies of the fusion sustaining component and dynamic features of the fusion initiating portion of the response. No statistically significant correlations were observed between either peak amplitudes or frequencies and the transient movement onset time, or the time at which maximum velocity occurred (p > 0.1). The spectral peaks are not related to time characteristics of the fusion initiating movement. However, a significant correlation assessed using the Pearson’s correlation coefficient (r = 0.61, p < 0.0001) was observed between the amplitude of the fundamental peak and the maximum velocity of the fusion initiating component for all responses from all subjects as shown in Figure 9.

## Discussion

Studies of eye movement dynamics provide insight into the neural mechanisms that drive these movements. For example, the high velocities seen in saccadic eye movements dictate that these movements must be driven by a large neural pulse in addition to a step signal. The oscillatory dynamics described here also provides insight into the control of sustained fusion. The frequency of these oscillations defines ranges of latency or delay in the feedback system and the amplitude relates to the gain, or amplification, of neural elements. The size of the harmonic peaks defines nonlinearities in the neural control system. Model simulations are required to fully exploit these measurements, but the values measured have direct implication on neural mechanisms of sustained fusion. Hence, the finding that the frequencies of fusion sustaining oscillations are much lower than initially reported and that the amplitudes are somewhat larger is of considerable importance as it places new constraints on elements in the feedback control system. These new constrains will be explored in a subsequent research involving new models and simulations.

If only the time domain representation of fusion sustaining vergence is analyzed, (for example, Figure 2, right-hand plots or Figure 3, left-hand plots), the small fluctuations observed could easily be dismissed as random drifts or other noise. However, in the frequency domain representation, the spectral plots show considerable structure with predominate fundamental frequency peaks followed by a series of harmonically related higher frequency peaks (summarized in Figures 6 and 7).

A similar general structure was found by Semmlow et al. (21) except that the frequency ranges and amplitudes were different due to differences in the amount of time recorded for eye movement response. They found fundamental frequencies ranging between 1.2 and 1.9 Hz and amplitudes ranging between 0.04 to 0.1 deg. Their average values for the amplitude and frequency of the fundamental frequencies correspond to the amplitude and frequency of the second or third harmonic peaks as shown here in Figure 6 and 7, respectively. This is likely due to the shorter time segments used in their study (1.2 sec or less). It is difficult to reliably identify oscillations that have oscillatory periods that are longer than the data segment available for analysis. The variability they found in fundamental frequencies was larger than in this study, possibly due to the fact that their fundamental frequency was actually a second harmonic in some subjects and a third harmonic in others. In light of their experience, it is not possible to unequivocally state that the fundamental frequencies found here are the lowest oscillatory frequencies in the fusion sustaining component. Eye movement recordings with longer fusion sustaining time periods would be required to definitively establish the fundamental frequencies and this is an area for future research.

Frequency peaks about the fundamental frequency, *f*_1_, may be harmonics of the fundamental oscillation as their values for increasing peak number, *n,* fall close to a straight line. This is particularly true of the middle three data points Figure 7 for all three convergence levels, Figure 5. However, the slopes of these lines should be equal to the fundamental frequency (Eq.1), but was found to be somewhat higher: 0.62 Hz as opposed to measured value of 0.47 Hz. Since the higher frequencies appear to be harmonically related (they fall very close to a straight line), we speculate that they are harmonics and that the fundamental frequencies measured are systematically lower than the actual fundamental frequencies. This may due to the difficulty of accurately measuring frequencies with periods close to the data length. Alternatively, we note that the standard deviations of frequency measurements were much larger as harmonic number increased, Figure 5. Hence, it is possible that the discrepancy in fundamental frequency is simply the product of measurement error. Finally, it is also possible that nonlinear features of the feedback control system may account for the discrepancy. Simulations of a model of the fusion sustaining vergence control system similar to the one described in Semmlow et al. (21) may explain the discrepancy.

Our findings are limited to sustained fusion at a vergence demand of 8.0 deg convergence that results from 4.0 deg. symmetrical convergence steps between 4.0 to 8.0 deg. Just as different levels of sustained convergence elicit different values of fixation disparity, it is likely that other levels of sustained convergence will show different oscillatory amplitudes and perhaps even a shift in frequencies. Variation in oscillatory features with the level of convergence is another topic of ongoing research.

Of particular interest is whether the preceding fusion initiating component has any influence on fusion sustaining oscillations. In other words, would the oscillatory behavior be the same if a specific convergence level were attained by a smaller convergence step, a divergent step, even a ramp stimulus, or no preceding step at all? Figure 9 shows a correlation between the average amplitude of the fusion sustaining fundamental frequency and the average maximum velocity of the fusion initiating component. This relationship is potentially the result of both the fusion initiating and fusion sustaining components sharing some common neural pathways; for example, the final common pathway and the oculomotor plant. Inter-subject variations in the gain of these common pathways would be reflected in the speed of the fusion initiating component as well as the amplitude of the oscillations in the fusion sustaining component. However, this correlation does not suggest a causal relationship between the two components. Again, a study of oscillatory behavior found at different levels of vergence and following a variety of step sizes would resolve this question.

The previous study of fusion sustaining oscillations included simulation of a model of this component’s probable feedback control elements. Data presented here show that this model is no longer valid at least with regard to the values of model elements. A major revision of this model is required in light of the new values of peak amplitude and frequency.

### Conclusion

The fusion sustaining component of vergence eye movements contains oscillations that have fundamental frequencies falling within a narrow range around 0.45 Hz. All subjects who exhibited higher frequency peaks that were probably harmonics of the fundamental frequency, but if so, they predict a higher fundamental frequency of approximately 0.62 Hz. The amplitudes of the fundamental frequency had a broader range than frequency varying between 0.04 and 0.18 deg rms. A correlation was found between fundamental frequency peak amplitude and the maximum velocity of the fusion initiating movement, probably the result of shared neural pathways. Additional studies are required to determine if the fusion initiating component has any influence on fusion sustaining oscillations. Further studies are also needed to determine the influence of convergence level on the oscillations. Nonetheless, the finding of consistent, well-defined fusion sustaining oscillations across a number of subjects provides very strong support for visual feedback control of sustained vergence.

The methods developed here can also be applied to clinical populations with vergence anomalies such as convergence and divergence insufficiency and excess. Prior research has shown that patients with convergence insufficiency have greater fixation disparity compared to binocularly normal controls (Saladin, 1986) which is the ability to reduce error within a feedback-controlled system. Yet, a study of the fusion sustaining component using frequency spectrum analyses has not yet been conducted on patients with vergency dysfunctions and is an area for future research as is the effectiveness of vision therapy on the behavior of the fusion sustaining component.
